# Fruit juices – more like fruit or sugar-sweetened beverages? Report of a symposium sponsored by the Fruit Juice Science Centre at IUNS-ICN Paris

**DOI:** 10.1017/jns.2025.10071

**Published:** 2026-01-06

**Authors:** Katie M. Hutchins, Carrie H.S. Ruxton

**Affiliations:** 1 Centre for Nutrition, Exercise and Metabolism, University of Bath, Bath, UK; 2 Department for Health, University of Bath, Bath, UK; 3 Nutrition Communications Cupar, UK

**Keywords:** Fruit juice, Fruit matrix, Glycaemic response, Sugars, Vascular function

## Abstract

A sponsored symposium was held at the International Congress on Nutrition to discuss the role of the fruit matrix in modulating the impact of 100% fruit juice (FJ) on markers of glycaemic control and vascular health and to present two recent studies. Structural, nutrient, and non-nutrient components of FJ, which comprise the fruit matrix and include polyphenols, pectins, vitamins, and minerals, have been shown in previous studies to influence postprandial metabolic responses. While the free sugar content of FJ and sugar-sweetened beverages (SSBs) can be similar, the fruit matrix distinguishes FJ from SSBs, the latter typically lacking in micronutrients and containing added sugars. Epidemiological studies consistently report that higher polyphenol intakes are associated with a lower risk of CVD, while some randomised controlled trials on citrus juices (rich in the flavanone, hesperidin) find beneficial effects for vascular function and blood pressure. Other randomised controlled trials report that FJ has neutral effects on cardiometabolic markers, which may be due to intra-individual differences in the digestion and absorption of polyphenols. The symposium concluded that the benign influence of the fruit matrix justifies the categorisation of FJ as a type of processed fruit, and not an SSB, for public health and regulatory purposes.

## Graphical abstract

**Figure:**
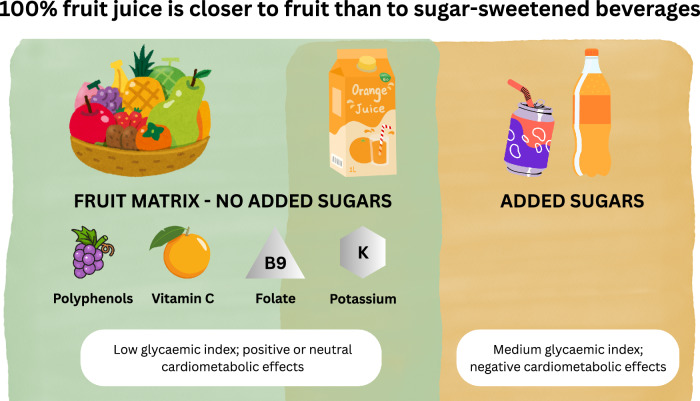

## Introduction

Pure fruit juice (FJ) contributes to intakes of vitamin C, folate, and potassium in European populations despite being consumed in relatively modest amounts.^(1,2)^ Mean intakes in consumers are 86 g/d for infants, 108g/d for children, 112 g/d for teenagers, and 135 g/d in adults.^(1)^ The inherent sugar content of FJ – which cannot contain added sugars, preservatives, colours or flavours by law^(3)^ – has created inconsistencies in public policy, resulting in some dietary guidelines placing FJ in the fruit category while others place it alongside sugar-sweetened beverages (SSBs). The polarisation of opinion about whether FJ is a type of processed fruit or an SSB has made the interpretation of scientific evidence on FJ and health outcomes challenging. This was the backdrop to a sponsored symposium at the International Union of Nutrition Sciences (IUNS) congress in Paris, which hosted three speakers, two of whom reported the findings of new studies. The aim of this report is to provide a summary of the symposium and contribute to future discussions on where FJ should sit within dietary guidelines.

## The fruit juice matrix, why it matters

The first speaker, Professor Javier Gonzalez, from the Centre for Nutrition, Exercise and Metabolism at the University of Bath, discussed the role of the FJ matrix in the post-ingestive metabolism of FJ. The matrix is defined as the complex structural and chemical environment of a food, its components (e.g. nutrients and non-nutrients), and how they interact. In the case of FJ, the matrix may include vitamins, minerals, pectin, carotenoids, and polyphenols, and has been reported to influence digestion, absorption, and metabolic responses. Several studies report that the human physiological response to the naturally occurring sugars in FJ differs from that of sugars added to SSBs – which may be explained by the FJ matrix.

FJ makes relevant contributions to nutrient intakes in the population, providing 16% of total polyphenol intake^(4)^ as well as vitamin C, potassium, folate, and vitamin A,^(5)^ whereas SSBs provide little nutritional value. When comparing the sugar content of SSBs and FJ, if expressed per 100 g, the total sugar content of SSBs and FJ is relatively similar, averaging approximately 10 g per 100 g in both categories. However, it is important to consider that the typical portion sizes consumed in the EU and the UK are different for these foods. An average portion of SSB (330 ml) typically provides ∼35 g of added sugars,^(6,7)^ whereas an average portion of FJ (150 ml)^(1)^ provides about 12–15 g of sugars, comprised of 25% glucose, 25% fructose, and 50% sucrose.^(6)^ In addition, the mean glycaemic index value (GI) of SSBs is generally higher (∼63), compared with that of FJ, which is typically lower (∼48)^(6,8)^. The lower GI value may partly be explained by the amount and type of sugars in FJ. A small (catalytic) dose of fructose has been shown to reduce the glycaemic response to a glucose load in animal models due to the impact on hepatic glucose metabolism. A previous study tested this effect in healthy human participants by administering a 75 g oral glucose tolerance test (OGTT) either with or without a 7.5 g fructose dose. The area under the curve for plasma glucose was significantly less (19%) in the OGTT + fructose condition when compared to the standard OGTT – fructose condition.^(9)^ This effect may be explained by a proposed mechanism in which low-dose fructose enhances hepatic glucose metabolism via glucokinase translocation, thereby increasing hepatic glucose uptake, glycolysis, and glycogen storage.^(9)^

There are several other ways the fruit matrix could modify metabolic control following ingestion of FJ, in particular due to the high polyphenol content. One of the polyphenols in citrus juices, hesperidin, may attenuate intestinal fructose and glucose absorption via partially inhibiting specific glucose transporters (GLUT2 and GLUT5), thereby reducing postprandial glycaemic responses. However, this effect may be modulated by the sugar and hesperidin concentrations.^(10)^ Another way by which the polyphenol content of FJ may act on glucose control is through increased urinary excretion of glucose. A randomised control crossover trial investigated this via administering polyphenol-rich apple extract (2.8 g) 30 min prior to an OGTT in 10 lean healthy men. Each participant completed a control OGTT (75 g glucose dissolved in 250 ml of water) for comparison. Ingestion of the apple extract prior to the OGTT was shown to increase renal glucose losses, with higher amounts of glucose detected in the urine over the 3-h period after the OGTT. This is thought to be due to inhibition of SGLT1 and/or SGLT2 in the kidney tubules preventing reabsorption of glucose^(11)^ therefore having favourable effects for glucose control.

FJ is often assumed to be fibre-free; however, it contains small amounts of pectin. The amount may vary according to the processing method and type of juice, for example, whether it is clear or cloudy or whether it contains pulp.^(6)^ Juices produced from pulping the whole fruit retain some of the fibre, whereas filtered juices contain minimal amounts.^(12)^ Although the fibre content of FJ is lower than that of whole fruit, consumption of 100% FJ has been associated with improved overall diet quality and higher intake of fruits and vegetables, thus also indirectly contributes to greater fibre intakes.^(13)^ The molecular structure of dietary fibres influences their physiochemical properties, which determine solubility, viscosity, and fermentability.^(14)^ These properties, in turn, affect their functionality and behaviour in the gastrointestinal tract, potentially modifying the rate of gastric emptying and intestinal transit.^(15,16)^ Such effects may slow glucose absorption and thereby attenuate postprandial glycaemic response.^(6,17,18)^

## Deciphering the true effect of the sugars in orange juice on postprandial glycaemia: relevance of other juice components and of interindividual differences

The next speaker, Professor Francisco Tomás-Barberán, from the Spanish National Research Council (CEBAS-CSIC), has long-standing research interests in the metabolism of plant bioactive compounds and their relevance to nutrition and health. Fruits and vegetables are rich sources of bioactive phytochemicals, including (poly)phenols. These have been linked to a wide range of protective effects against oxidative stress and inflammation, both of which play key roles in the development of cardiometabolic diseases.^(19)^ Although (poly)phenolic compounds have a relatively low absorption from the small intestine, they reach the colon where they undergo intensive metabolism by the microbiota.^(20)^ There is increasing evidence to suggest that (poly)phenols and derived compounds (both endogenous and microbiota-derived metabolites) can exert biological activity by functioning as signalling molecules which may influence gene expression, modulate enzyme activity, regulate epigenetic mechanisms, and engage in crosstalk with the gut microbiota. Collectively, these actions can contribute to the regulation of vascular and metabolic processes, including nitric oxide bioavailability, redox balance, inflammatory responses, insulin sensitivity, and lipoprotein function.^(21)^ In one trial, healthy participants consumed orange juice (OJ) that had been diluted two-fold (i.e. 100 ml OJ mixed with 100 ml water) with added hesperidin (49 mg per 200 ml portion, approximately 0.5 mM total). Postprandial blood glucose levels were lower after this high hesperidin-diluted OJ compared with a matched control drink that contained equivalent amounts of sugars, citric acid, malic acid, and ascorbic acid as the OJ, but no added hesperidin. This study design therefore allowed the specific effects of hesperidin to be distinguished from the components of the juice (i.e. juice matrix).^(10)^ The authors hypothesised that hesperidin could modulate the postprandial glycaemic response to sugars via partial inhibition of intestinal glucose transporters (GLUT). Hence, bioactive (poly)phenols present in the OJ, such as hesperidin, would be expected to influence the absorption and metabolism of the natural sugars in the juice. Importantly, by implementing a metabolomics approach, Professor Tomás-Barberán and his team at CEBAS have shown substantial variability in the absorption and excretion of citrus bioactive compounds, influenced by gut microbiota activity and enzymatic differences.^(22)^ Thus, more intervention studies in humans are needed to understand the glycaemic response to the intake of FJs such as OJ, the influence of other components in the juice such as the (poly)phenols, as well as to investigate the variability in the responses.

Along these lines and building on the first presentation, Professor Tomás introduced the ‘ZULEMA’ intervention trial, which randomised healthy men in a crossover design to various test drinks containing the same content in sugars as in the OJ but with different proportions of other nutrients and (poly)phenols naturally present in the OJ. The main aim of this project was to compare the postprandial glycaemic responses between the drinks, with an insight into the interindividual variability and metabolomic differences. The ZULEMA results are currently under review for publication.

## Fruit polyphenols and vascular health – Christine Morand

The third speaker, Dr Christine Morand from the French National Institute for Agriculture, Food and Environment (INRAe), leads a research group investigating the role of diet and plant food bioactives in vascular health. Her presentation focused on dietary polyphenols, with an emphasis on their bioavailability and role in CVD risk. She also presented preliminary findings from the ongoing ‘HESPER health’ study, which investigated the effects of hesperidin on vascular function.

Fruits and FJ are important sources of dietary polyphenols, providing an extensive range of compounds in biologically relevant amounts. The mean intake of polyphenols among a cohort of 4942 French adults was estimated at 1193 ± 510 mg/d or 1.3 g/d based on 24 h dietary records. Non-alcoholic beverages, including coffee, tea, and FJ, were identified as major sources of dietary polyphenols, accounting for over half of daily intakes (658 ± 426 mg/d), with coffee contributing 79%, tea 17%, and juice 2%.^(23)^ The most common polyphenols in European diets belong to the flavonoid family (with flavanols, flavanones, flavonols, anthocyanins as major representatives), and phenolic acids (mainly hydroxycinnamic acids and ellagitannins). Ingested polyphenols are significantly transformed before entering the circulation, and their structural differences influence intestinal absorption and bioavailability. A small proportion (5–10%) is absorbed in the upper part of the intestine and undergoes conjugation in the intestine and liver, while the majority (90–95%) reaches the colon, where, prior to their absorption, they are metabolised by gut microbiota into diverse phenolic acids and bioactive metabolites. Poorly absorbed polyphenols may still have local effects in the gut (e.g. antioxidant, anti-inflammatory, modulation of microbiota), and their microbial metabolites may drive some of the known systemic health benefits.^(24)^

The relationship between polyphenol intake and cardiovascular health has been well studied. Prospective cohort studies provide strong evidence for associations between polyphenol intake and cardiovascular health. A meta-analysis of thirty-nine prospective cohorts reported that a higher total flavonoid consumption was associated with a reduced incidence of CVD.^(25)^ Randomised controlled trials investigating the vascular effects of FJ have reported mixed results. For OJ, some studies reported reductions in blood pressure and improvements in endothelial function, with effects more consistently observed with blood OJ.^(26–32)^

Hesperidin, a flavanone glycoside abundant in OJ, is a proposed key mediator of these effects. In a randomised crossover trial in healthy, overweight men, a 4-week supplementation with OJ significantly lowered diastolic blood pressure compared with placebo. In addition, both OJ and hesperidin supplementation in a control drink improved postprandial microvascular endothelial reactivity, thus supporting a potential causal role for hesperidin.^(29)^ In a 6-month randomised controlled trial (RCT), postmenopausal women consumed 0.3 L/d of grapefruit juice naturally rich in naringin, or a matched control drink without this flavanone compound.^(33)^ A significant reduction in arterial stiffness was observed only with the grapefruit juice, highlighting a specific benefit of naringin.

Blueberry anthocyanins may also influence vascular function, as suggested by a 12-week supplementation trial in healthy older adults, which found a significantly reduced 24 h systolic blood pressure compared with placebo.^(34)^ Inconsistencies across studies may reflect variation in FJ doses, polyphenol content, and heterogeneous study populations, highlighting the need for greater standardisation in future trials.

Positive correlations between improvement in vascular outcomes and the plasma levels of fruit polyphenol metabolites have been observed across several trials,^(35–37)^ highlighting the role of these compounds in mediating the vascular protective effects of fruits and indicating that the bioavailability and metabolism of polyphenols are key determinants of their effectiveness. The biological plausibility for the vascular protective effects of plasma polyphenol metabolites is increasingly documented. For example, in experimental models, the circulating phase-2 flavanone metabolites have been shown to enhance vasodilation, via increasing nitric oxide-mediated vasodilation,^(38,39)^ exert anti-inflammatory effects via suppression of the NF-kB pathway and reduction of iNOS/COX-2 expression,^(40)^ and improve vascular integrity by reducing monocyte adhesion and modulating endothelial gene expression.^(41)^

The HESPER health trial^(42)^ is a three-arm, blinded, crossover RCT in healthy, slightly overweight middle-aged men with the aim of investigating the vascular effects of moderate OJ consumption and establishing the role of hesperidin. Participants consumed either 330 ml/d of OJ, an isoenergetic SSB, or the same SSB supplemented with hesperidin for a 6-week period. The results are currently being prepared for publication.

It is widely accepted that leveraging fruit polyphenols is a promising strategy in dietary prevention of CVD, and moderate intakes in healthy individuals may still induce meaningful molecular changes, which suggests their long-term potential in public health advice.

## Conclusion

To conclude, whole fruits, FJ, and SSBs differ in fibre, vitamins, minerals, and polyphenols, all of which can exert different effects on physiological processes and metabolism. The fruit matrix, especially polyphenols, appears to have a significant ameliorating effect on the body’s response to consuming fruit sugars and may positively influence markers of health. Hence, it is more rational to categorise FJ as a processed fruit rather than an SSB. Furthermore, available evidence does not seem to support the exclusion of FJ from dietary guidelines.
